# Short-interval total femur replacement for infected distal femoral reconstruction

**DOI:** 10.1093/jscr/rjag122

**Published:** 2026-03-07

**Authors:** Danil Chernov, Nicholas Frappa, Morgan Dillon, Alexander Kovacs, Matthew Alben, Ryan Riley, Joseph B Kuechle

**Affiliations:** Jacobs School of Medicine and Biomedical Sciences, University at Buffalo, 955 Main St. Buffalo, NY 14203, United States; Jacobs School of Medicine and Biomedical Sciences, University at Buffalo, 955 Main St. Buffalo, NY 14203, United States; Jacobs School of Medicine and Biomedical Sciences, University at Buffalo, 955 Main St. Buffalo, NY 14203, United States; Department of Orthopaedics and Sports Medicine, University at Buffalo, 462 Grider St., Buffalo, NY 14215, United States; Department of Orthopaedics and Sports Medicine, University at Buffalo, 462 Grider St., Buffalo, NY 14215, United States; Department of Orthopaedics and Sports Medicine, University at Buffalo, 462 Grider St., Buffalo, NY 14215, United States; Department of Orthopaedics and Sports Medicine, University at Buffalo, 462 Grider St., Buffalo, NY 14215, United States

**Keywords:** short-interval revision, total femur replacement, periprosthetic joint infection, distal femur, replacement, osteosarcoma

## Abstract

We present the case of a 61-year-old woman, initially treated for distal femoral osteosarcoma with a distal femur replacement (DFR) and multiple prior revisions, who presented with chronic infection of the DFR and catastrophic proximal femoral bone loss, representing a formidable reconstructive challenge. She underwent a novel short-interval two-stage revision consisting of proximal femur resection and placement of a custom antibiotic-loaded total femur spacer, followed 1 week later by definitive total femur replacement. Early follow-up demonstrated infection control and preservation of a functional limb. This case highlights the potential of an abbreviated two-stage approach for limb salvage in complex periprosthetic infections with extensive bone loss.

## Introduction

Limb-salvage procedures, including distal femoral replacement (DFR), have significantly improved the quality of life for patients undergoing treatment for osteosarcoma [1, 2]. However, DFR is associated with a high incidence of complications in this already vulnerable oncologic population. Periprosthetic joint infection (PJI) remains a particularly challenging complication, compounded by risk factors, such as prior radiation therapy and complex limb reconstruction [3].

When complications occur, reconstructive options are limited. Total femur replacement (TFR) offers a salvage strategy in cases of severe bone loss and infection, providing structural continuity between the hip and knee [4]. Traditionally, the treatment of PJI involves a two-stage revision with long intervals of 6–12 weeks between stages and prolonged spacer retention [5]. Although serological and synovial biomarkers often aid in guiding the timing of reimplantation, there are currently no accurate metrics that reliably predict outcomes [6]. In a prospective cohort of 38 patients, short-interval protocols of 4 weeks or less demonstrated no significant difference in infection eradication or clinical outcomes compared with long-interval protocols [7].

We present the case of a 61-year-old woman initially treated with distal DFR for osteosarcoma, who developed PJI, proximal femoral bone loss, and implant failure. The infection was successfully managed with a two-stage conversion to total femoral replacement with a 1-week interval. At 1 year follow-up, there were no signs of clinical or radiographic implant failure or PJI. To our knowledge, this represents one of the shortest reported successful intervals for a total femur antibiotic spacer in an oncologic setting.

## Case report

**Figure 1:**
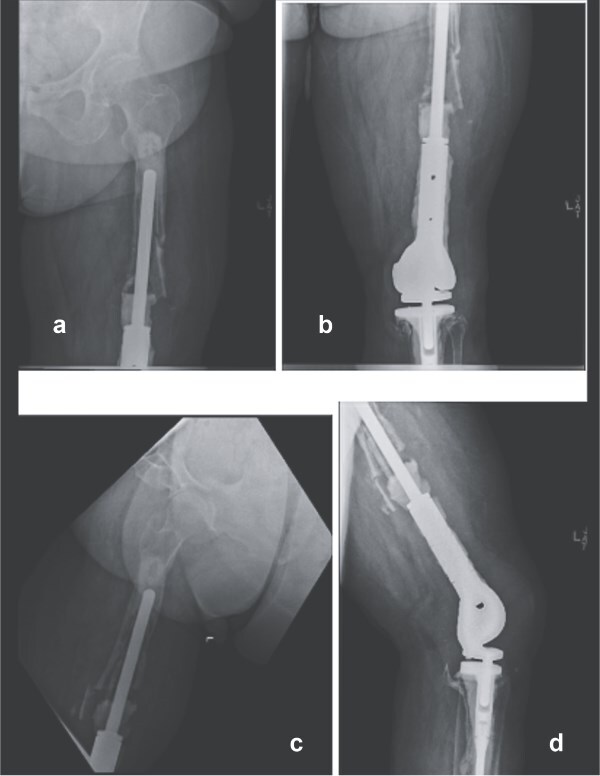
Preoperative anteroposterior (a and b) and lateral radiographs (c and d) of the right femur showing loosening of the proximal femoral stem and extensive proximal femoral bone loss following multiple distal femoral replacement revisions for osteosarcoma.

A 61-year-old woman with a history of osteosarcoma treated with DFR who presented with recurrent infection, extensor mechanism loss, and progressive proximal femoral bone loss. She had undergone multiple failed revisions and debridements. Radiographs showed loosening of the proximal femoral stem with minimal remaining viable femoral bone (Fig. 1). Given the extent of bone loss and infection, a two-stage revision with TFR was planned.

### Stage one

**Figure 2:**
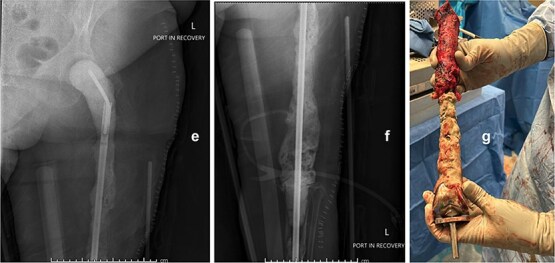
Intraoperative anteroposterior radiographs (e and f) after stage one, demonstrating a custom antibiotic-loaded total femur spacer constructed using an Ilizarov rod embedded in antibiotic cement and a fully cement-coated fusion nail extending from the acetabulum to the tibia. The resected right femoral implant is grossly visualized (g) prior to spacer placement.

Following implant removal and thorough debridement, a custom total femur antibiotic spacer was fashioned using an Ilizarovrod embedded in antibiotic-loaded cement, coupled to a fully cement-coated fusion nail (Fig. 2). This construct extended from the acetabulum to the tibia, preserving limb length and joint space. The patient was kept non-weight-bearing and initiated on intravenous cefazolin. The postoperative course was uneventful, with no immediate complications.

### Stage two

Seven days later, reimplantation was performed. A press-fit cup with a dual mobility liner was placed in the acetabulum to reduce dislocation risk. A modular TFR system was used: proximally, a femoral stem compatible with the dual mobility head; distally, a rotating-hinge knee mechanism attached to a tibial stem. Soft-tissue reconstruction was performed as feasible. The wound was closed over a drain and protected with a negative-pressure dressing. The patient remained non-weight-bearing with a knee immobilizer and continued intravenous cefazolin postoperatively for six weeks.

**Figure 3:**
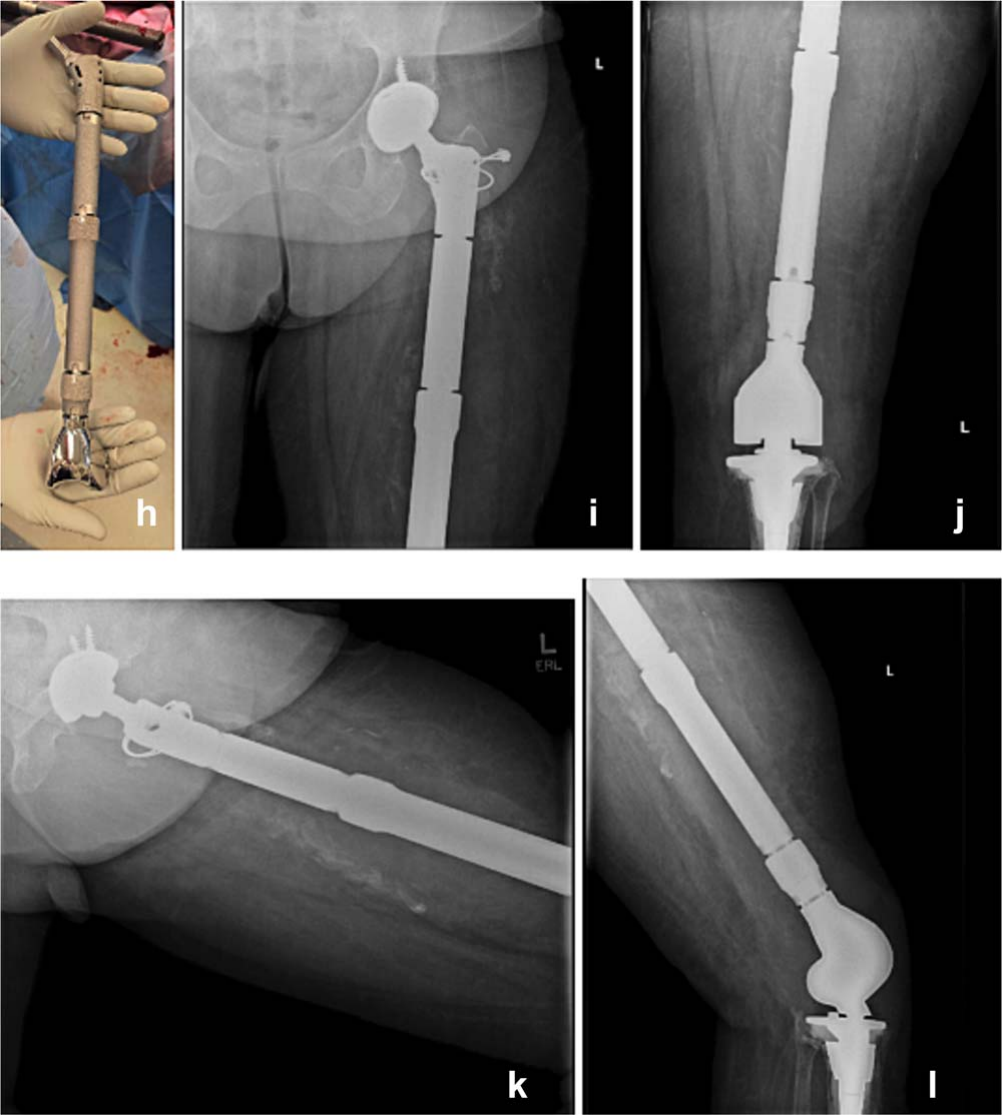
The modular TFR implant (h) is grossly visualized prior to implantation. Immediate postoperative anteroposterior (i and j) and lateral (k and l) radiograph following definitive reimplantation, showing the modular total femur replacement with a dual mobility acetabular component proximally and a rotating-hinge knee mechanism distally in appropriate alignment.

Postoperatively, early imaging confirmed appropriate implant alignment. Motor function was preserved, and the incision healed without complication. She began a structured rehabilitation program with gradual progression to protected ambulation using an orthotic knee brace. At follow-up, the patient demonstrated functional limb

use, no signs of recurrent infection, and a stable prosthesis without evidence of hardware failure (Fig. 3).

**Figure 4:**
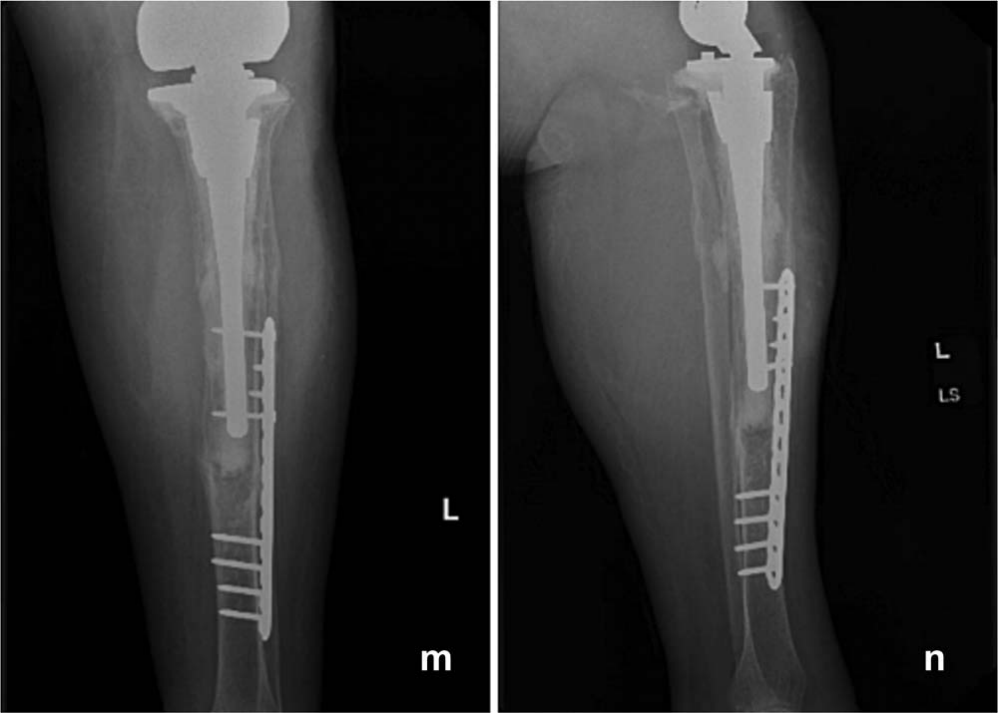
Follow-up anteroposterior (m) and lateral (n) radiographs at 1 year demonstrating intact tibial fixation following fracture repair and a stable total femur construct without evidence of loosening or reinfection.

At 6 months postoperatively, the patient sustained a tibial shaft fracture following a pivoting injury while exiting her vehicle. She underwent operative fixation with a plate and was managed initially in a splint, followed by transition to a controlled ankle motion (CAM) boot. At 1 year follow-up from index procedure, she continued to progress well, with intact fixation and a stable total femur construct (Fig. 4). Although this subsequent injury was unrelated to the index procedure but is included for completeness and transparency in her clinical course.

## Discussion

Periprosthetic joint infection following distal femoral replacement represents one of the most challenging complications in limb-salvage orthopedic oncology. These cases are often complicated by polymicrobial infection, prior radiation, and extensive bone loss, all of which limit reconstructive options [8]. When the femur becomes unsalvageable, TFR remains a viable strategy to restore limb length and function [4].

In the present case, we used a short-interval two-stage revision to treat infection and catastrophic proximal femoral bone loss. This approach deviates from the traditional two-stage protocol that typically employs a 6–12 interval between procedures. Our shortened interval was designed to reduce patient immobility, minimize soft-tissue contracture, and enable earlier mobilization while maintaining infection control through targeted intravenous cefazolin. Our experience supports previous findings showing that short-interval protocols can achieve infection eradication rates comparable to longer spacer durations [7].

Successful limb salvage in this setting requires meticulous debridement, adequate antibiotic delivery, and stable reconstruction. High-dose antibiotic cement and thorough debridement ere critical to early infection control, consistent with outcomes from larger TFR spacer series in which 92% of patients were successfully reimplanted and 75% remained infection-free at 3 years [9]. Favorable intraoperative findings and clean surgical conditions supported our decision to proceed with reimplantation after only 1 week.

Implant selection is central to achieving stability and function in these complex reconstructions. Dual mobility acetabular components have been shown, in high-risk revision settings, to reduce the risk of dislocation [10], while rotating-hinge knee mechanisms provide varus-valgus and anteroposterior stability to compensate for deficient soft tissues [11]. In this case, combining a dual mobility hip with a hinged knee offered maximal joint stability and allowed for early functional recovery despite extensive soft-tissue compromise.

This case highlights that a short-interval two-stage conversion from distal femoral reconstruction to total femur replacement can achieve both infection eradication and limb preservation in select patients. Although long-term follow-up is required, our results suggest that an abbreviated interval may reduce morbidity and maintain function without compromising infection control, provided that surgical and antimicrobial principles are rigorously applied.
